# Ovarian adenosarcoma in a postmenopausal woman: Case report and review of literature

**DOI:** 10.1016/j.ijscr.2023.108244

**Published:** 2023-04-23

**Authors:** Azam sadat Mousavi, Narges Zamani, Mona Mohseni, Fatemeh Zamani, Sedigheh Ghasemian Dizaj Mehr, Soheila Sarmadi

**Affiliations:** a Department of Gynecologic Oncology, Vali-Asr Hospital, Tehran University of Medical Sciences, Tehran, Iran; b Department of Obstetrics and Gynecology, Women's Hospital, Tehran University of Medical Sciences, Tehran, Iran; c Department of Radiology, Children Medical Center of Excellence, Tehran University of Medical Sciences, Tehran, Iran; d Department of Obstetrics and Gynecology, School of Medicine, Urmia University of Medical Sciences, Urmia, Iran; e Pathology, Tehran University of Medical Sciences, Tehran, Iran

**Keywords:** Adenosarcoma, Case report, Postmenopausal, Sarcomatous overgrowth

## Abstract

**Introduction and importance:**

Mullerian adenosarcoma is a rare malignancy that generally occurs in the uterine corpus but uncommonly, it may be found extrauterine. Ovarian adenosarcoma is extremely rare and often is presented in reproductive age women. Most of them are low grade and have à good prognosis except for adenosarcoma with sarcomatous overgrowth.

**Case presentation:**

A 77-year-old menopausal woman presented with abdominal discomfort. She had severe ascites and elevated levels of CA-125, CA 19-9, and HE4 tumor markers. Adenosarcoma with sarcomatous overgrowth was diagnosed after the histopathological examination of the surgical biopsy.

**Conclusion:**

The possibility of endometriosis transformation to malignancy even in postmenopausal women may warrant continuous follow-up for early diagnosis of ovarian cancer, this potentially fatal disease. More studies are needed to find the best therapeutic approach to adenosarcoma with sarcomatous overgrowth.

## Highlights

• Müllerian adenosarcoma can arise in both uterine and extra uterine locations.
• Adenosarcoma from endometriosis can recognized in menopause.
• There has been suspicion of origin of extra-uterine adenosarcoma from endometriosis.

## 1 Introduction

Adenosarcoma is a rare malignancy from the Mullerian mixed tumors group. It typically originates from the uterine corpus, but uncommonly, it may be found in the cervix and ovary and may arise outside the genital tract [1]. Ovarian adenosarcoma is extremely rare and represents <1 % of ovarian malignancies [2]. The etiology of adenosarcoma is unknown; however, the correlation between extra uterine adenosarcoma and endometriosis is reported in some studies [3]. The typical symptom of uterine adenosarcoma is abnormal uterine bleeding in postmenopausal women, although it was also observed at a younger age. Extra-uterine adenosarcoma does not exhibit a specific feature and often presents with abdominal discomfort [4], [5]. Excluding adenosarcomas with sarcomatous overgrowth with a poor prognosis and considerable risk of recurrence, the overall prognosis of adenosarcoma is good [6]. In contrast to their uterine counterpart, ovarian adenosarcomas are more aggressive with a poor prognosis, usually present in the advanced stage and young patients [4], [7]. The studies about ovarian adenosarcoma are only case reports or series.

Eichhorn et al. found that only 40 cases of ovarian adenosarcoma were reported until 2002 in their comprehensive literature review [4]. We will present a rare case of extra-uterine adenosarcoma in a 77-year-old menopausal woman. It is unexpected to encounter adenosarcoma of the ovary arising from endometriosis in late post-menopause. We will discuss these pathologic challenging features and educational points raised from this case study.

Written informed consent was obtained from the patient to publish this case report and accompanying images. A copy of written consent is available for review by the Editor-in-Chief of this journal on request. This work has been reported per the SCARE 2020 criteria [8].

## 2 Case presentation

**Fig. 1:**
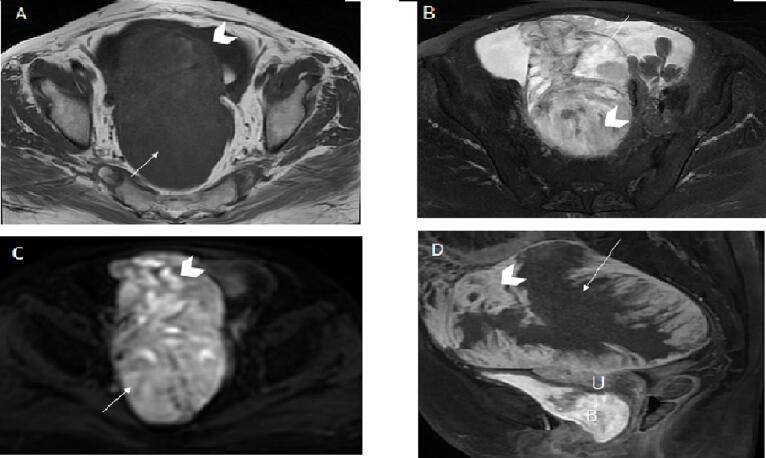
The cystic component (arrow) shows slight hyperintensity on axial T1WI (A), homogeneous hyperintensity on axial T2WI with fat suppression (B), no enhancement on contrast-enhanced T1WI with fat suppression (C), isointensity on DWI (D). The mural nodule (arrow head) demonstrates isointensity (A), heterogenous hyperintensity (B), marked enhancement (C) and hyperintensity on DWI (D). U: uterus, B: bladder.

A 77-year-old multiparous (all with Natural Vaginal Delivery, NVD) was referred to the clinic complaining of generalized abdominal pain. She had menopause at 50 and no family history of the disorder or receiving medications, except for her comorbid illnesses, if any. On review of the system, she declared suffering from dysmenorrhea during her reproductive years of life, but she neither was followed up nor visited a gynecologist for a check-up. On abdominal examination, mild to moderate ascites was found. In pelvic and abdominal ultrasonography, severe ascites in the abdominopelvic cavity and the thickening of the omentum were noted. A large 155 ∗ 87 mm solid cystic mass with internal vascularity in the pelvic cavity was reported. The mass seemed to originate from the right adnexa. The left ovary was atrophic. An abdominal and pelvic Magnetic Resonance Imaging (MRI) study was done, which revealed a mainly cystic mass with a large mural nodule [Fig. 1].

A unilateral, oval-shaped, large mass with 171 ∗ 102 ∗ 87 dimension on the posterior right side of the uterus was seen, which was mainly cystic with mural nodules, a cystic component with homogeneous isohyperintensity on T1WI, and marked enhancement in mural nodules.

Ascites and omental thickening were visualized. The right fallopian tube was thick and irregular. Adhesion of ileal loops to mass was depicted. Advanced ovarian cancer was the first differential diagnosis.

Hematologic examination showed the following results: WBC (white blood cell): 9300, Hgb (hemoglobin): 11.9, Plt (platelet): 499,000, BHCG (B human chorionic gonadotropin): negative, LDH (lactate dehydrogenase): normal, CA125 (cancer antigen 125): 740 U/mL (normal range = 0–35), CA19-9 (carbohydrate antigen 19-9): 78 U/mL (normal range = 0–37), and HE4 (human epididymis protein 4): 262 pmol/L (normal range < 140). The patient has undergone laparoscopic surgery in another center, and a biopsy of the mass was done in addition to peritoneal cytology submitted for study. The pathology result was not inclusive and reported only inflammation and necrosis. The patient was referred to our center and underwent laparotomy surgery. Severe inflammation, ascites, and adhesion of the tumor and intestine were found intraoperatively. There was evidence of endometrial implants. This amount of adhesion and inflammation in the patient without past surgical history probably indicated underlying undiagnosed endometriosis. Total hysterectomy and bilateral salpingo-oophorectomy were done. Supracolic omentectomy, partial resection of the involved intestine with re-anastomosis, was done during the adnexal surgery. Peritoneal cytology samples were sent to the laboratory. The pathological report of surgical samples was as follows:

‐ Cytology was negative for malignancy.
‐ The cervix, endometrium, and myometrium were normal, but the tumor involved serosal layers.
‐ Left adnexa was negative for malignancy
‐ Right adnexa had low-grade endometrioid stromal sarcoma with glandular differentiation.
‐ No residue of the ovary was seen.
‐ The tumor was infiltrated around adipose tissue. Extensive foci of necrosis were identified.
‐ No lymphovascular invasion was seen.
‐ The right fallopian tube and omentum were negative.
‐ The intestine sample showed serosal involvement by the tumor.

IHC (Immunohistochemistry) study of the sample revealed positive Vimentin, CD10 (cluster of differentiation 10), WT1 (Wilms tumor 1), ER (estrogen receptor), and PR (progesterone receptor) on tumoral cells. Hence, the diagnosis of low-grade endometrioid stromal sarcoma was confirmed for the patient.

The level of CA125, one month after surgery, decreased to 13 and became negative.

Three courses of chemotherapy were prescribed with the Paclitaxel and Carboplatin regimen. Radiotherapy was done for her. Follow-up MRI studies were done at the end of chemotherapy sessions, during radiotherapy, and four months after surgery. The MRI report was as follows:

- There are four solid structures. The largest one is 77 ∗ 62 mm in the right paracolic gutter, and the other lesions are in the pelvic cavity.
- Contrast enhancement and abnormal restriction are noted in solid portions of the mentioned lesion.

Chest CT scan was normal.

**Fig. 2:**
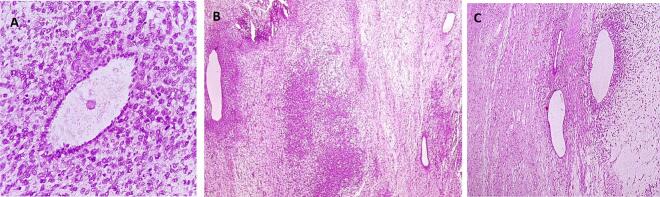
(A): ×400 H&E staining feature (B), (C): ×100 H&E staining feature.

The pathology sample was reassessed and reviewed again, and the following new findings were reported [Fig. 2]:

Although there were no significant pathologic changes in theuterus, omentum, and left adnexa, but Right adnexa was adenosarcoma with sarcomatous overgrowth

- Sarcomatous overgrowth was poorly differentiated/undifferentiated sarcoma
- Mitotic rate: 51 in 10 HPF (high power field)
- Extra ovarian involvement was presented probably a serosal layer of the intestine.
- Lymph vascular invasion: Not identified.
- Necrosis: present, extensive
- Tumor tissue on small bowel resection:

Marked fibroblastic reaction, inflammatory cell infiltration, and focuses suggestive of non-cystic endometriosis were seen.

- The serosal layer of the intestine was involved in the tumor.

According to the new pathology result, the chemotherapy regimen with iphosphomide and doxorubicin was started for the patient.

After three months, our patient was followed by MRI and tumor markers. Fortunately, compared with previous imaging, the number and size of the lesions decreased. In response to chemotherapy, tumor markers level became low. In the last follow-up (two years after the recurrence), she survived with good performance, and the disease was stable, according to imaging studies.

## 3 Discussion

Malignant mesenchymal neoplasms account for 1-3% of all female genital tract tumors, and Mullerian Adenosarcoma (MA) constitutes 8-10% of these malignancies. Mullerian adenosarcoma is an uncommon tumor consisting of two stromal and epithelial parts described in 1974 [4]. From the histopathologic point of view, the epithelial portion represents a benign glandular endometrioid, but the stromal component is usually low-grade malignant. The differential diagnosis of MA with sarcomatous overgrowth also includes Endometrial Stromal Sarcoma (ESS), immature teratoma, Malignant Mullerian Mixed Tumors (MMMT), and pure sarcomas when heterologous elements are present. ESS occurs in the same age group as mullerian adenosarcoma, is usually unilateral, and resembles a stromal component of adenosarcoma but lacks its glandular component. Thorough sampling should be done to search for the epithelial components of MA, as the prognosis of MA is worse than that of ESS [9].

It is a common belief among expert pathologists that microscopic morphologic features usually make a histologic diagnosis of adenosarcomas, although challenging. Therefore, reviewing the slides by an expert gyneco-pathologist could lead to the final diagnosis. Immunohistochemical staining is not vital for the demonstration of sarcomatous nature. Adenosarcoma is mainly found in the uterine; however, extra-uterine involvement in the ovary, cervix, and peritoneum has been reported [1], [4]. Our report presents a case of extra-uterine adenosarcoma involving the ovary and intestine. Usually, uterine adenosarcomas are diagnosed in postmenopausal women, but ovarian adenosarcomas tend to involve younger patients. Eichhorn et al. reviewed all the reported cases of ovarian adenosarcomas that were 40 patients until that time. They found that most reviewed cases were under 50 years (48 %) [4]. Patients are typically early postmenopausal, with the usual signs and symptoms of an adnexal mass [10]. In contrast to the finding of this review, our patient was a 77-year-old lady in menopause. The disease presentation differs according to the sites of involvement [9]. In uterine adenosarcoma, the most common symptom is abnormal vaginal bleeding, but the major findings in extra uterine adenosarcomas are abdominal pain, discomfort, and mass. Mandato et al., in a literature review in 2018 on 41 patients with primary extra-uterine, extra-ovarian Mullerian adenosarcoma, reported abdominal or pelvic pain as the primary presentation in 14 out of 34 (41 %) symptomatic patients. In Eichhorn et al. study, abdominal mass was detected in half of the patients during physical examination [4], [5]. Compatible with these studies, our patient's initial presentations were abdominal pain and distention. The etiology of adenosarcoma is undetermined; however, its correlation with endometriosis in extra uterine cases is explained as a possible cause [3]. Previous studies reported a range of 11 to 61 % of correlation with endometriosis, which was higher in extra uterine-extra ovarian cases [4], [5]. In contrast to most studies that demonstrated the correlation between endometriosis and adenosarcoma at young ages, our case presented such a correlation in older age.

AS is the second most common gynecological malignancy in patients with endometriosis after clear cell carcinoma of the ovary [6]. Endometriosis transformation to adenosarcoma in menopausal women without hormonal therapy was an interesting point in our patient. The presence of adhesion and conclusive pathological finding for endometriosis confirmed the above-mentioned correlation. Sampson first defined the following criteria for diagnosing malignant transformation of endometriosis: i) There should be a clear example of endometriosis in proximity to the tumor, ii) no other primary site of the tumor may be found, and iii) the histological appearance should be consistent with an endometrial origin [7]. In the present case, the transition between endometriosis and adenosarcoma was confirmed to meet Sampson's criteria [7].

Ovarian adenosarcomas are typically low-grade but sometimes accompany sarcomatous overgrowth [1]. Despite the overall good prognosis, detecting sarcomatous overgrowth increases aggressiveness, and higher risks of recurrence and metastasis are expected [9]. Ovarian adenosarcoma also warrants a worse prognosis than its uterine counterpart [4], [10]. There are multiple explanations for this worse prognosis. The location of the tumor in the abdominal cavity, absence of an anatomical barrier, larger size, higher stage of the tumor at presentation, and significant risk of tumor rupture can lead to a poor prognosis [4]. A better prognosis was reported for correlated adenosarcoma and endometriosis [5]. Our patient suffered from ovarian adenosarcoma with sarcomatous overgrowth and intestinal involvement. Early recurrence occurred four months after the adnexal surgery. Extensive foci of necrosis and a mitotic rate of 51 in 10 HPF were detected. All these findings indicated a poor prognosis for our patient. A literature review has shown that the rate of SO in MA ranges from 8 % to 54 % [1], [2], [3], [4], [5], [6], [7], [8], [9], [10], [11], [12], [13], [14], [15], [16]. A study conducted by Gallardo and Prat. [13] determined SO in 18 (33 %) cases, and the mitotic index varied from less than one to 30 per 10 HPF (mean: 3/10 HPF). To our knowledge, this high mitotic rate in ovarian adenosarcoma was undescribed before in the literature. Shakuntala et al. 2012 mentioned the rarity of elevated CA125 in adenosarcoma [14]. Inoue et al. considered the elevated CA125 a probable indicator of sarcomatous overgrowth [15]. Eleven out of 41 patients (all extra uterine-extra ovarian adenosarcomas) reviewed by Mandato had an increased level of CA125, with two having a simultaneously high level of CA125 and CA19-9 [5].

Our patient's laboratory data showed higher-than-normal values for CA125, CA19-9, and HE4. To our knowledge, this is the first case of ovarian adenosarcoma with an elevated level of HE4. The levels of tumor markers came down to normal ranges after surgery. In a case report by Hirakawa et al., an ovarian adenosarcoma was described that had ascites with positive cytology [16]. Most ascites correlating with ovarian adenosarcoma represent negative cytology [14]. Our patient's cytology was negative for malignancy despite severe ascites in the abdominopelvic cavity, confirming that the cytological study does not help diagnose adenosarcoma. The recurrence rate of uterine adenosarcoma is about 25 % compared to a 50 % recurrence rate in ovarian adenosarcoma. The mortality risk of ovarian adenosarcoma exceeds 35 %, considerably higher than that of uterine adenosarcoma. The low incidence of Mullerian adenosarcomas complicates the development of well-defined and consensual action protocols. The main goal of adenosarcoma treatment is radical surgery and optimal debulking. Achieving this goal seems hard in ovarian adenosarcoma [5]. Adjuvant therapies like chemotherapy, radiotherapy, and hormonal therapy are also described for this situation in the literature. The lack of powerful clinical trials warrants more evaluations for approving the role of adjuvant therapy [5]. We did optimal surgery for the patient, resulting in good condition, but the patient experienced early recurrence despite receiving adjuvant therapy. We revised the pathological study, finding adenosarcoma with sarcomatous overgrowth dictates us to change the chemotherapy regimen. After two years of the recurrence, she survived, and the disease was stable.

Increased levels of CA125 and HE4 tumor markers, presence of ascites, and malignant transformation of endometriosis to adenosarcoma with sarcomatous overgrowth in postmenopausal women are among the interesting points in this patient.

## 4 Conclusion

The possibility of endometriosis transformation to malignancy, even in postmenopausal women, may warrant continuous follow-up for early diagnosis of this potentially fatal disease. We believe annual gynecologist check-ups are important in women's primary care. More studies are needed to find the best therapeutic approach to adenosarcoma with sarcomatous overgrowth. Considering that in the last 40 years, >100 cases have been reported in the literature [4], [11], a worldwide registry is urgently needed to collect data regarding these rare AS to standardize treatment and obtain reliable data on prognosis.

## Consent

Written informed consent was obtained from the patient for publication of this case report and accompanying images. A copy of the written consent is available for review by the Editor-in-Chief of this journal on request.

## Ethical approval

Not applicable.

Ethical approval is exempt/waived at our institution.

## Funding

Nothing to declare.

## Author contribution

A.S.M.: Main surgeon of the patient, Editing the final manuscript.

N.Z.: writing and editing the article, corresponding.

M.M.: collecting data.

F.Z.: reporting and interpretation of patient's imaging.

S.G.D.M.: collecting data.

S.S.: preparing pathology figures.

## Guarantor

Narges Zamani.

## Research registration number

N/A.

Not applicable.

## Provenance and peer review

Not commissioned, externally peer-reviewed.

## Declaration of competing interest

Nothing to declare.
